# Clinical Epidemiology of Buruli Ulcer from Benin (2005-2013): Effect of Time-Delay to Diagnosis on Clinical Forms and Severe Phenotypes

**DOI:** 10.1371/journal.pntd.0004005

**Published:** 2015-09-10

**Authors:** Carlos Capela, Ghislain E. Sopoh, Jean G. Houezo, René Fiodessihoué, Ange D. Dossou, Patrício Costa, Alexandra G. Fraga, João F. Menino, Rita Silva-Gomes, Edgard M. Ouendo, Fernando Rodrigues, Jorge Pedrosa

**Affiliations:** 1 Life and Health Sciences Research Institute (ICVS), School of Health Sciences, University of Minho, Braga, Portugal; 2 ICVS/3B’s—PT Government Associate Laboratory, University of Minho, Braga/Guimarães, Portugal; 3 Buruli Ulcer Treatment Center of Allada, Allada, Bénin; 4 Regional Institute for Public Health, Ouidah, Bénin; Fondation Raoul Follereau, FRANCE

## Abstract

Buruli Ulcer (BU) is a neglected infectious disease caused by *Mycobacterium ulcerans* that is responsible for severe necrotizing cutaneous lesions that may be associated with bone involvement. Clinical presentations of BU lesions are classically classified as papules, nodules, plaques and edematous infiltration, ulcer or osteomyelitis. Within these different clinical forms, lesions can be further classified as severe forms based on focality (multiple lesions), lesions’ size (>15cm diameter) or WHO Category (WHO Category 3 lesions). There are studies reporting an association between delay in seeking medical care and the development of ulcerative forms of BU or osteomyelitis, but the effect of time-delay on the emergence of lesions classified as severe has not been addressed. To address both issues, and in a cohort of laboratory-confirmed BU cases, 476 patients from a medical center in Allada, Benin, were studied. In this laboratory-confirmed cohort, we validated previous observations, demonstrating that time-delay is statistically related to the clinical form of BU. Indeed, for non-ulcerated forms (nodule, edema, and plaque) the median time-delay was 32.5 days (IQR 30.0–67.5), while for ulcerated forms it was 60 days (IQR 20.0–120.0) (p = 0.009), and for bone lesions, 365 days (IQR 228.0–548.0). On the other hand, we show here that time-delay is not associated with the more severe phenotypes of BU, such as multi-focal lesions (median 90 days; IQR 56–217.5; p = 0.09), larger lesions (diameter >15cm) (median 60 days; IQR 30–120; p = 0.92) or category 3 WHO classification (median 60 days; IQR 30–150; p = 0.20), when compared with unifocal (median 60 days; IQR 30–90), small lesions (diameter ≤15cm) (median 60 days; IQR 30–90), or WHO category 1+2 lesions (median 60 days; IQR 30–90), respectively. Our results demonstrate that after an initial period of progression towards ulceration or bone involvement, BU lesions become stable regarding size and focal/multi-focal progression. Therefore, in future studies on BU epidemiology, severe clinical forms should be systematically considered as distinct phenotypes of the same disease and thus subjected to specific risk factor investigation.

## Author Summary

Buruli Ulcer (BU) is a neglected disease caused by *Mycobacterium ulcerans*. Clinical presentations of BU lesions are classically classified as papules, nodules, plaques and edematous infiltration, ulcer or osteomyelitis. Within these different clinical forms, lesions can be further classified as severe forms based on focality (multiple lesions), lesions’ size (>15cm diameter) or WHO Category (WHO Category 3 lesions). There are studies reporting an association between delay in seeking medical care and the development of ulcerative forms of BU or osteomyelitis, but the effect of time-delay on the emergence of lesions classified as severe has not been addressed. To address both issues, and in a cohort of laboratory-confirmed BU cases, 476 patients from a medical center in Allada, Benin, were studied. In our cohort, we validated previous observations, demonstrating that time-delay is statistically related to the clinical form of BU, namely ulcers and osteomyelitis. However, time-delay is not related with more severe phenotypes, implying that severe clinical forms of BU should be considered as distinct phenotypes of the same disease and subjected to specific risk factor investigation.

## Introduction

Buruli ulcer (BU), caused by *Mycobacterium ulcerans*, is the third most common mycobacteriosis worldwide, after tuberculosis and leprosy [1]. BU pathogenesis is mediated by mycolactone, a potent polyketide-derived macrolide that triggers apoptotic cell death [2] and is associated with the necrotic nature of the disease [3]. BU mostly affects people in tropical countries in Africa [4], America [5], Asia [6] and Australia [7]. Although no official estimate of global incidence is available at present, West Africa is the main endemic area, with 1967 new cases reported by Côte d'Ivoire, Ghana, and Benin in 2013[8]. BU is a devastating necrotising skin infection characterised by pre-ulcerative lesions (papules, nodules, plaques and edematous infiltration), which commonly develop into ulcers with undermined edges and can spread to an entire limb [9] and can also affect the bone (osteomyelitis) [10]. Moreover, within these clinical presentations, more aggressive severe forms of BU, such as multiple lesions, larger lesions or higher World Health Organization (WHO) categories have been described [11], although underreported and less understood. Epidemiological studies on *M*. *ulcerans* transmission, on BU risk factors and on the host immune status, suggest that the variable frequency of BU and its distinct clinical forms are related to: i) age; ii) gender; iii) preferential anatomical site; iv) water contact; and v) regional occurrences [12,13,14,15,16].

To date, a reduced number of risk factors underlying the severe BU phenotypes had been reported. HIV co-infection is one of the few examples. Some studies revealed an increased BU prevalence among HIV patients, especially those presenting large lesions and osteomyelitis [17,18]. Specifically, low CD4 cell counts were significantly associated with larger lesions and patients with a CD4 cell count below 500 cell/mm^3^ took twice as long to recover from BU when compared with individuals with a normal CD4 cell count [19]. Other risk factors, such as hypoproteinemia [11] and anemia [20] were also identified to be associated with severe forms of BU disease.

In addition, the delay in seeking medical care and the late medical diagnosis of BU have been proposed to account for the disease presentation [21,22,23,24]. In fact, in BU endemic regions the culture and beliefs are powerful factors that affect proper medical intervention, as patients preferentially seek treatment from traditional practitioners, or herbalists [22]. On top of this, the lack of knowledge on the available treatments and their effectiveness, the financial constraints during hospitalisation, fear of treatment, and poor access to health facilities are also important aspects delaying the pursuit of proper treatment [25,26]. Indeed, delay in seeking medical care has been previously associated with the distinct BU clinical forms. Taking into consideration that the time from progression of a pre-ulcer to an ulcer is variable and can range from a few weeks to several months (e.g. estimated average time of 30–90 days) [27], it was established that individuals with non-ulcerated forms had a median delay of 30 to 45 days, while individuals with ulcers presented a 60-day delay and patients with osteomyelitis up to 90 days [28]. Thus, the more advanced and destructive ulcerated forms and osteomyelitis are associated with longer delay-periods, while non-ulcerated forms are more common in patients with recent infection [28], justifying the importance of early diagnosis and treatment for the disease.

Nonetheless, more aggressive, severe clinical presentations of BU, such as large lesions (>15cm in diameter) and multifocal lesions, have also been described [11], although the underlying pathological mechanisms are yet unclear [29]. While this can be associated with characteristics of the patient itself (genetic susceptibility/health status) or with the virulence of the infecting strain, it is also rational to question the influence of the delay in health seeking on the appearance of the more severe forms of BU. To our knowledge, the latter aspect is yet to be studied. Therefore, to uncover whether the time-lapse between the first remembered symptoms and clinical diagnosis is associated to disease severity, we retrospectively analysed a cohort of 476 laboratory-confirmed BU treated cases discovered in a highly endemic area in Allada, Benin, between 2005 and 2013.

## Materials and Methods

### Ethics statement

Ethical approval (clearance Nu 018, 20/OCT/2011) for integrating studies on BU was obtained from the National Ethical Review Board of the Ministry of Health in Benin, registered under the Number IRB0006860. The *Centre de Dépistage et de Traitement de l'Ulcère de Buruli* (CDTUB)—Allada and the national BU control program authorities approved access to the registry. All data analyzed in this study was anonymized.

### Study setting, participants and design

We retrospectively collected clinical data from 476 laboratory-confirmed BU patients of CDTUB in Allada, Benin—between January 2005 and December 2013. At the moment of diagnosis, parameters such as age, gender, major clinical form (nodule, plaque, edema, ulcer or osteomyelitis) and multifocal presentations were registered. For mixed clinical forms, the most severe lesion was considered the major clinical form. Additionally, lesion size (cm, considering major diameter), WHO category [30] (Category 1: maximum lesion diameter <5cm; Category 2: maximum lesion diameter 5–15cm; and Category 3: minimum lesion diameter >15cm associated or not with osteomyelitis and/or multifocal lesions and/or at a critical site), lesion site (upper or lower limb, trunk, head and/or neck) and laboratory confirmation tests (culture of *M*. *ulcerans* from the lesion, histopathology with the presence of acid-fast bacilli, or highly specific IS2404 real-time PCR) were taken into consideration. The HIV status was also retained for the present study and excluded from analysis if positive.

Delay in seeking medical care (time between first symptoms or signs remembered and medical attendance) was also recorded. The time of seeking medical care was defined as the moment of diagnosis and treatment initiation. All included patients completed antibiotherapy according to the WHO recommendations and were treated with surgical procedures [30].

### Statistical analysis

Explanatory and descriptive analysis of the study cohort was performed based on the following variables: age at the moment of the BU diagnosis; gender; clinical form (ulcer, plaque, edema, nodule and osteomyelitis); lesion site; and lesion severity. Severe phenotypes were defined as multifocal lesions (more than one lesion); large lesions (diameter >15cm) or Category 3 lesions (minimum lesion diameter >15cm associated or not with osteomyelitis,multifocal lesions and/or at a critical site) as classified by the WHO recommendations. Median comparisons were performed through one-way ANOVA’s (Brown-Forsythe and Welch, when applicable) using age, gender, site of lesion, clinical BU form; and lesion severity as explanatory variables and time-delay seeking medical care (days, using means and medians distribution in each group) as a dependent variable. Unadjusted and adjusted (for age-cutoff value 15 years of age- and gender binary or linear logistic regression models) odds ratios were then calculated to explore the effects of time-delay in diagnosis into the clinical form of BU lesions, and particularly into severe phenotypes of BU. We systematically fit the model, controlling age (dichotomized or ordinal) and gender with the considered time-delay (to seek medical attendance) as explanatory variables for each of the clinical lesions and severe phenotypes defined for BU. All the described analyses were obtained using IBM SPSS Statistic v. 22. A result was considered significant for p<0.05.

## Results

### Cohort characterization

The BU cohort (CDTUB, Allada, Benin), comprising 476 cases, had laboratory BU confirmation by at least one laboratory diagnostic test, as recommended by the WHO. Results were positive for IS2404 RT-PCR in 430 (90.3%) cases and Ziehl-Neelsen staining in 327 (68.7%) cases. All cases were HIV negative. The median age at diagnosis was 12 years (IQR: 7–24 years; mean 17.9 ± 16.3 years), with 321 (67.4%) patients 15 years old or under. Although the overall gender ratio of the patients was balanced (245 [51.5%] male) (Table 1), a major distortion of this ratio was recorded as a function of age, with males being predominant in younger patients and females in older patients (OR 2.99, 95%CI 2.00–4.46, p = 0.0001). Specifically, male patients accounted for 193 (60.1%) of the patients younger than 15, but only 52 (33.5%) of those were over 15 (Table 1).

**Table 1:** Age and gender distribution according to lesion location, lesion phenotype, and lesion severity in 476 laboratory-confirmed BU treated patients at CDTUB—Allada from 2005 to 2013.

	Patient n (%)	Age distribution (>15 | ≤15)	Gender distribution (F|M)
***Gender***			
Male	245 (51.5%)	52 | 193	..
Female	231 (48.5%)	103 | 128	..
***Age***			
> 15 years old	155 (32.6%)	..	103 | 52
≤ 15 years old	321 (67.4%)	..	128 | 193
***Lesion location*** ^a^			
Head and neck	6 (1.3%)	2 | 4	4 | 2
Thorax and abdomen	43 (9.0%)	9 | 34	18 | 25
Upper Limb	171 (35.9%)	51 | 120	88 | 83
Lower Limb	256 (53.8%)	93 | 163	121 | 135
Lower limb lesions vs. Upper limb lesions	256 (53.8%)	93 | 163	121 | 135
	220 (46.2%)	62 | 158	110 | 110
***Clinical lesion***			
Nodule ^a^	4 (0.8%)	0 | 4	3 | 1
Edema ^a^	24 (5.0%)	4 | 20	10 | 14
Plaque ^a^	125 (26.3%)	44 | 81	62 | 63
Ulcer ^a^	320 (67.2%)	105 | 215	155 | 165
Ostemyelitis ^a^	3 (0.6%)	2 | 1	1 | 3
All	476 (100%)	155 | 321	231 | 245
Non-ulcerative vs. Ulcerative forms	156 (32.8%)	49 | 107	77 | 82
	320 (67.2%)	105 | 215	155 | 165
Edema vs. Other non-ulcerated forms	24 (5.0%)	4 | 20	10 | 14
	129 (27.1%)	44 | 85	65 | 64
***Multifocal lesions***			
Multifocal	22 (4.6%)	8 | 14	10 | 12
Unifocal	454 (95.4%)	147 | 307	221 | 233
***Lesion size***			
> 15cm	142 (29.8%)	47 | 95	62 | 80
≤ 15cm	334 (70.2%)	108 | 226	169 | 165
***WHO Category***			
Category 3	161 (33.8%)	55 | 106	71 | 90
Category 1 + 2	315 (66.2%)	100 | 215	160 | 155

^a^ dominant clinical form

**Fig 1:**
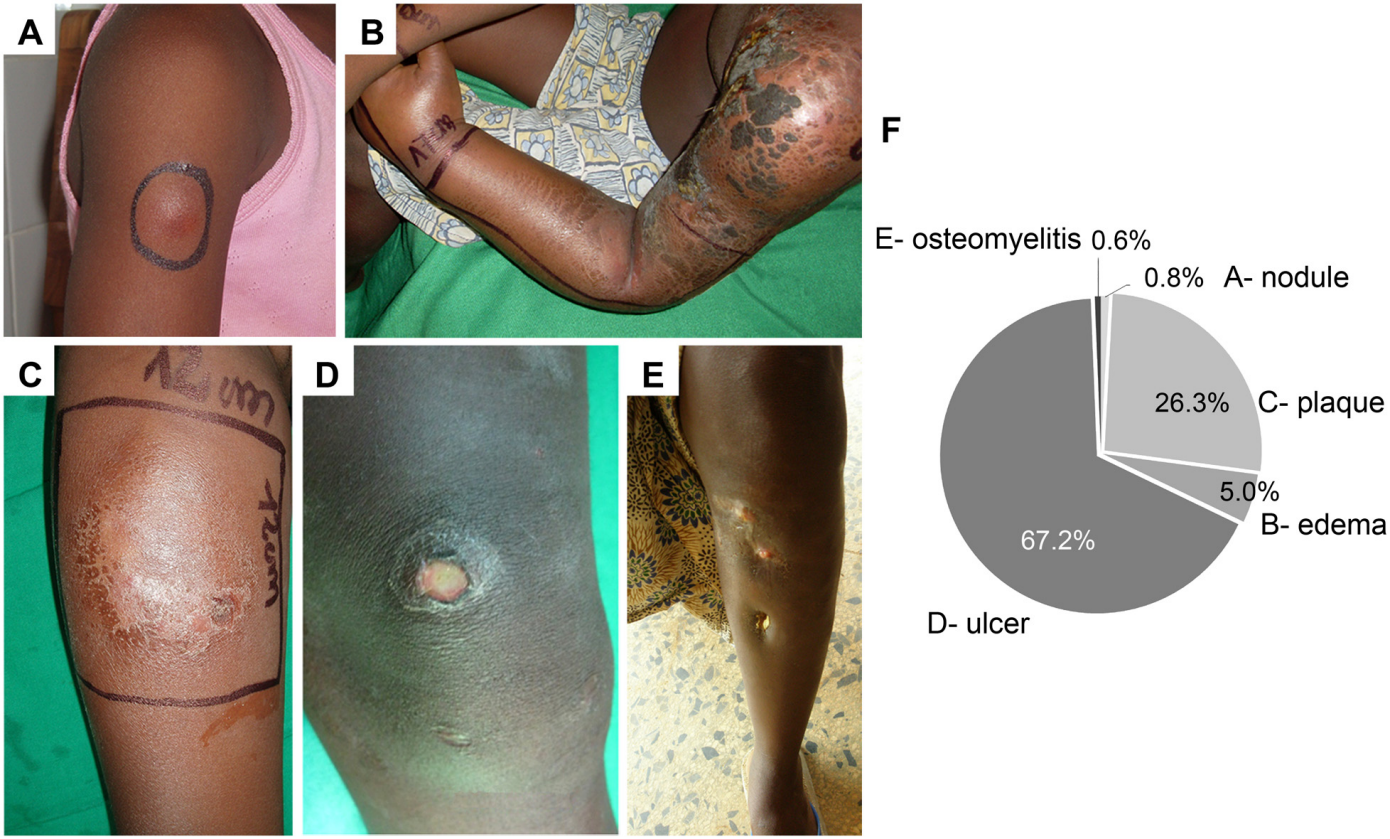
Prevalence of clinical BU lesions. Representative images of (A) nodule, (B) edema, (C) plaque, (D) ulcer, (E) osteomyelitis and (F) the percentage of each clinical presentation.

**Fig 2:**
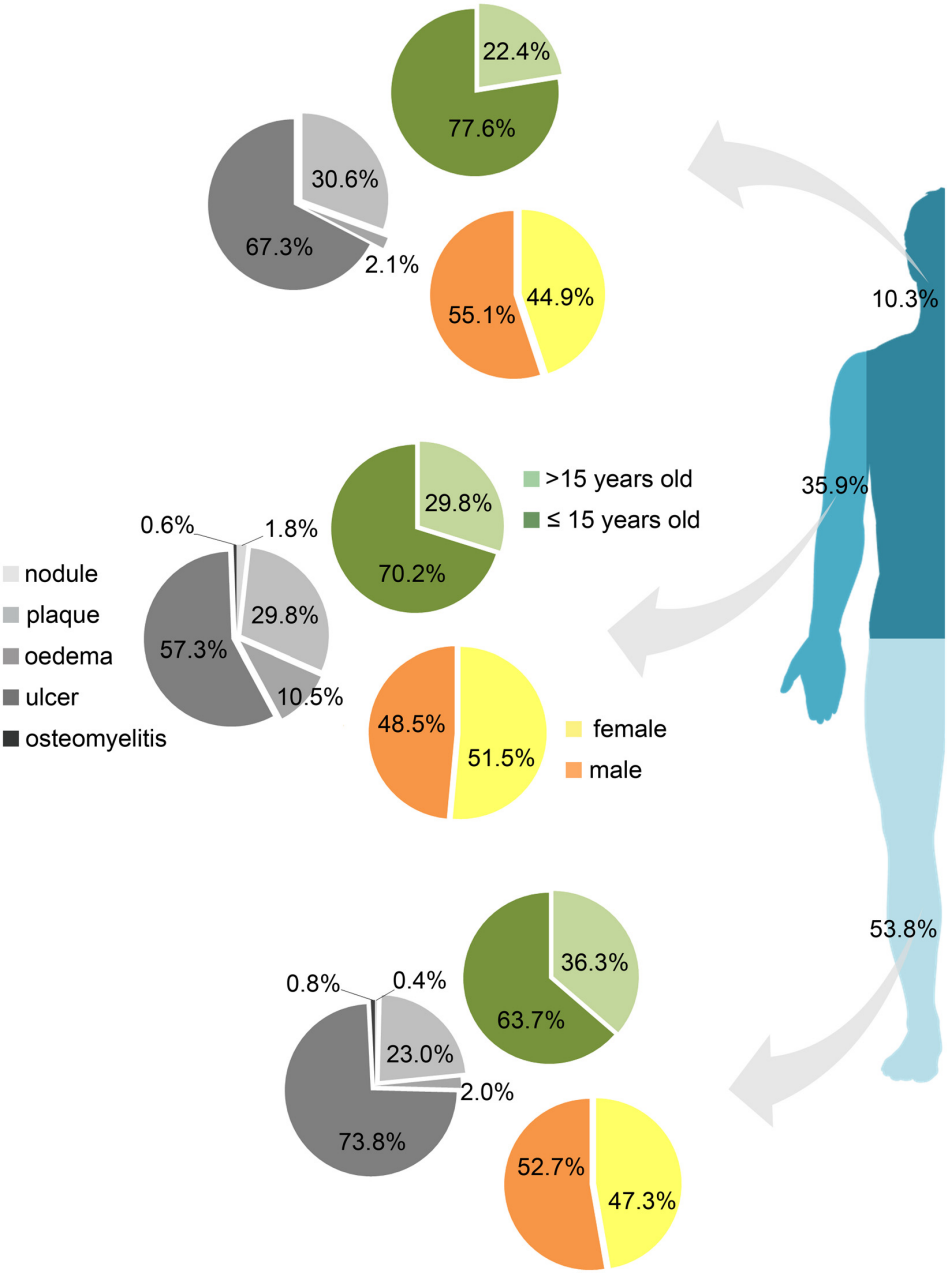
Age, gender, clinical BU lesion, and lesion location. Age (≤15 years old, >15 years old), gender, and clinical BU forms according to lesion distribution throughout the three major areas of the body (lower limbs, upper limbs, and head+trunk).

Considering the dominant clinical BU form per patient, 4 (0.8%) presented nodules (Fig 1A and 1F and Table 1), 24 (5.0%) presented edema (Fig 1B and 1F and Table 1), 125 (26.3%) presented plaques (Fig 1C and 1F and Table 1), and 320 (67.2%) presented ulcers (Fig 1E and 1F and Table 1). Osteomyelitis was diagnosed in 5 patients (1.1%), and was considered the most relevant form in 3 of the patients (0.6%) (Fig 1E and 1F and Table 1). Concerning the site of lesions, 256 (53.8%) patients presented lesions on the lower limbs, while 171 (35.9%) had lesions on the upper limbs (Fig 2 and Table 1). Atypical sites (head, neck and/or trunk) accounted for 49 (10.3%) patients (Fig 2 and Table 1). Site of the lesion and relative age, gender and dominant clinical form distribution are represented in Fig 2.

**Fig 3:**
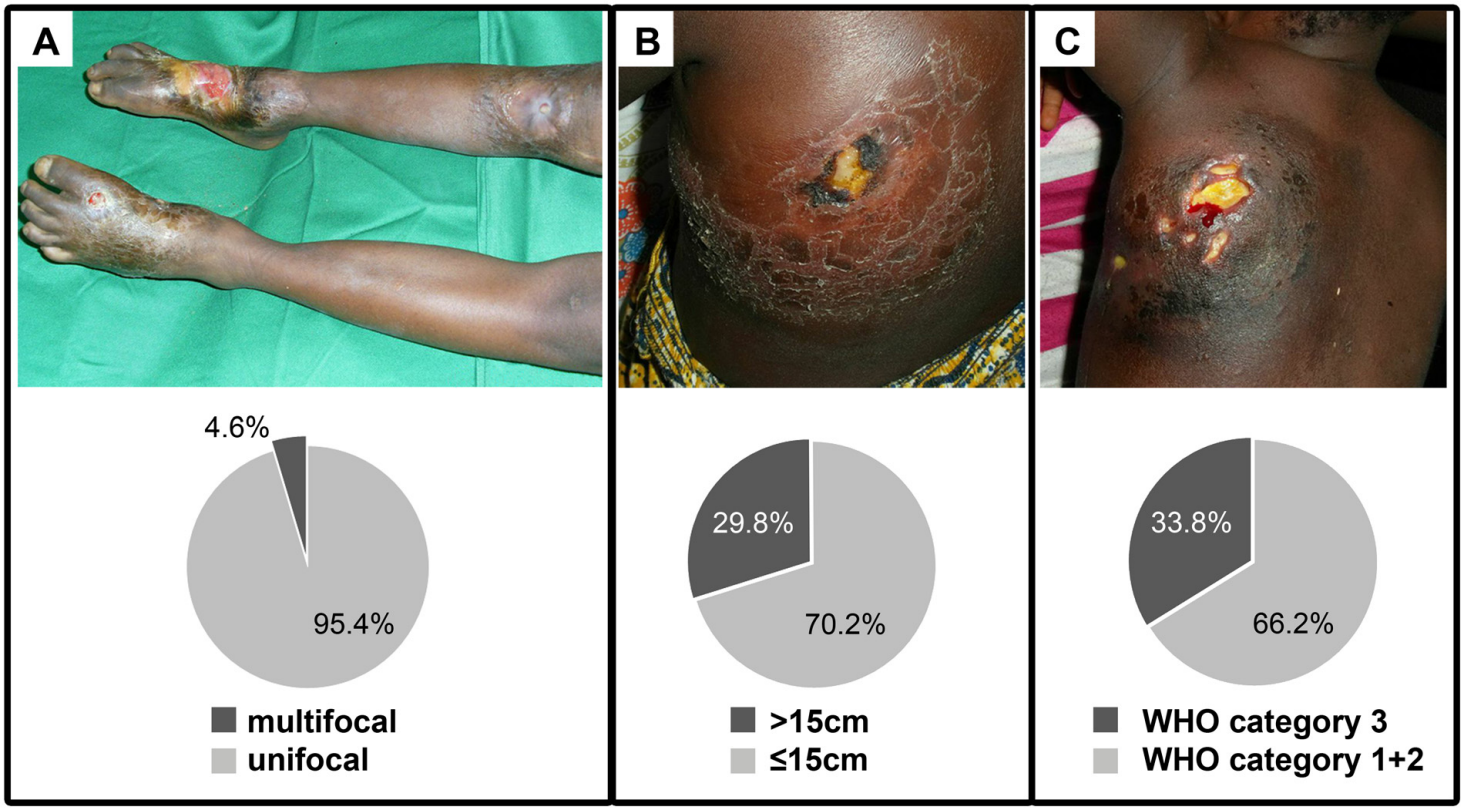
Prevalence of severe BU lesions. Representative images of (A) multifocal lesions, (B) large lesions (>15cm), (C) WHO Category 3 lesions, and the percentage of each clinical presentation.

Regarding the observed severe forms of BU, 22 (4.6%) patients presented lesions in more than one localization (Fig 3A and Table 1), while 142 (29.8%) patients presented lesions larger than 15cm in major diameter (Fig 3B and Table 1). The WHO category 3 is a broader classification given that it comprises patients with multiple lesions, lesions with a diameter >15cm associated or not with osteomyelitis and/or lesions at a critical site. Taking into account these criteria, we recorded 315 (66.2%) patients in category 1+2 and 161 (33.8%) in category 3 (Fig 3C and Table 1).

The different clinical presentations, as well as the severe forms of these lesions, were subjected to age and gender adjustments (Tables 2 and 3, respectively). No significant interference was recorded in the binary logistic regression, except for upper body lesions (upper limb, head or neck), for which there was an overrepresentation of younger ages (OR 0.986, 95%CI 0.974–0.998, p = 0.018) (Table 2).

**Table 2:** Univariate analysis of the effect of age and gender on clinical BU forms and lesion location in 476 laboratory-confirmed BU treated patients at CDTUB—Allada from 2005 to 2013 (binary logistic regression).

	Coeff. (SE)	Odds Ratio (CI)	p-value
***Male***			
Age	0.006	0.972 (0.960–0.984)	**0.0001**
***Upper body lesions*** ^a^			
Age	0.006	0.986 (0.974–0.998)	**0.018**
Gender	0.200	0.811 (0.559–1.177)	0.271
***Nodule*** ^b^			
Age	0.105	0.867 (0.706–1.065)	0.174
Gender	1.164	0.218 (0.022–2.135)	0.191
***Plaque*** ^b^			
Age	0.006	1.003 (0.991–1.016)	0.624
Gender	0.213	0.965 (0.635–1.465)	0.866
***Edema*** ^b^			
Age	0.020	0.970 (0.933–1.008)	0.116
Gender	0.433	1.136 (0.486–2.654)	0.768
***Ulcer*** ^b^			
Age	0.006	1.004 (0.992–1.016)	0.534
Gender	0.200	1.039 (0.702–1.537)	0.850
***Osteomyelitis*** ^b^			
Age	0.042	0.972 (0.896–1.055)	0.501
Gender	0.934	1.220 (0.196–7.603)	0.831

^a^ upper body lesions: head + neck + upper limbs + thorax + abdomen

^b^ dominant clinical form

**Table 3:** Univariate analysis of the effect of age and gender on severe BU forms in 476 laboratory-confirmed BU treated patients at CDTUB—Allada from 2005 to 2013 (binary logistic regression).

	Coeff. (SE)	Odds Ratio (CI)	p-value
***Multifocal lesions***			
Age	0.012	1.010 (0.986–1.035)	0.433
Gender	0.450	1.224 (0.507–2.956)	0.653
***Larger lesions (***>***15cm)***			
Age	0.006	1.004 (0.992–1.017)	0.489
Gender	0.207	1.363 (0.909–2.043)	0.134
***WHO category 3***			
Age	0.006	1.004 (0.992–1.016)	0.550
Gender	0.200	1.342 (0.908–1.985)	0.140

### Time-delay to seek medical care

**Fig 4:**
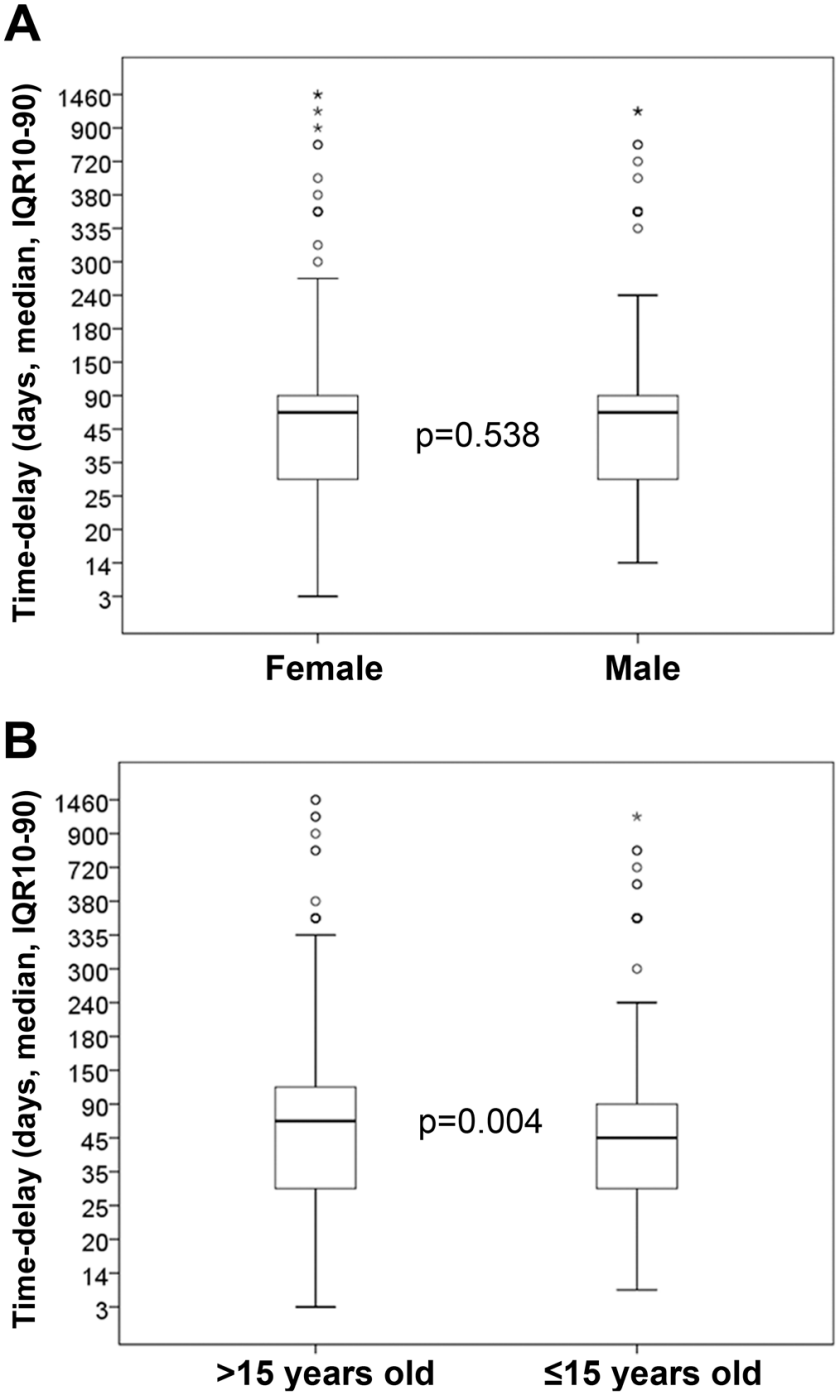
Time-delay to seek medical care related to gender and age. Time-delay to seek medical care related to (A) gender and (B) age. Circles represent the outliers and asterisks represent the extreme outliers. Statistical significance was calculated using Welch's *t*-test. Differences with a p-value of ≤0.05 were considered significant.

The overall mean time-delay to seek medical care was 101.1 days (95%CI 86.3–117.0) (S1 Table). Since the variable time-delay does not follow a normal distribution (Kurtosis = 48.2; Skewness = 6.4), median variations were considered to compare the distinct behavior of dependent variables. Time-delay to seek medical care was indistinct for male and female gender (p = 0.538) (Fig 4A and S1 Table): median was 60 days [IQR 30–90] for both genders. However, age was associated with significantly different delay times (p = 0.004) (Fig 4B and S1 Table). Median was 60 days [IQR 30–120] for patients over 15 years old at the moment of the diagnosis; while time-delay was 45 days [IQR 30–90] for patients with 15 years of age or under.

**Fig 5:**
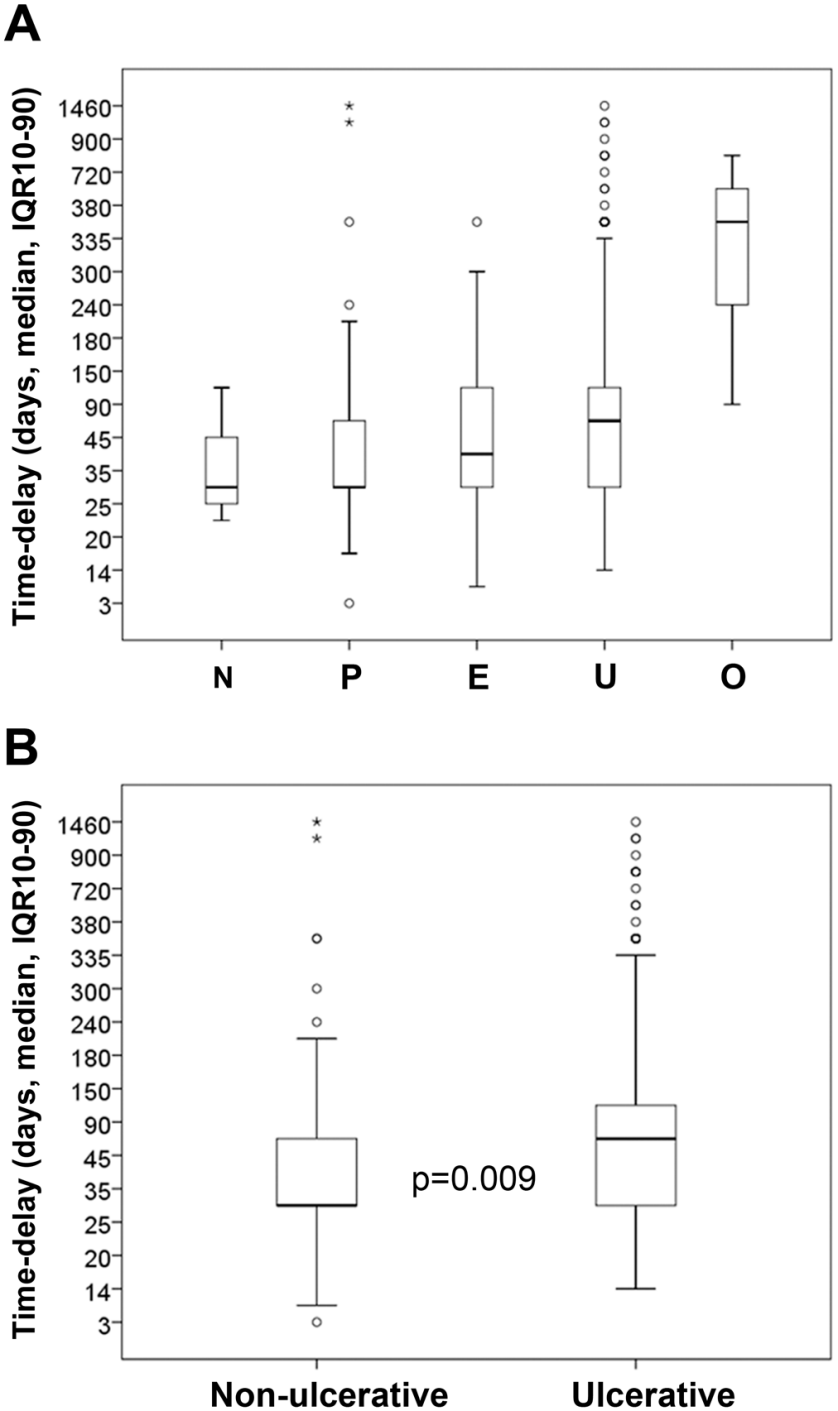
Time-delay to seek medical care related to clinical BU forms. Time-delay to seek medical care related to (A) clinical form N—nodule; P—plaque; E—edema; U—ulcer; O—osteomyelitis and (B) non-ulcerative vs. ulcerative lesions. Circles represent the outliers and asterisks represent the extreme outliers. Statistical significance was calculated using Welch's *t*-test. Differences with a p-value of ≤0.05 were considered significant.

Time-delay was also related to the clinical form of the disease (Fig 5). Median was 32.5 days [IQR 30–67.5] for non-ulcerated forms (nodule, edema, and plaque); 60 days [IQR 20.0–120.0] for ulcerated forms; and 365 days [IQR 228–548] for bone lesions. When the time-delay among patients with non-ulcerated versus ulcerated forms was compared, we confirmed significant discrepancies (p = 0.009) (Fig 5B and S1 Table). In addition, among the non-ulcerated clinical forms, edema was significantly associated with longer time-delays when compared with others non-ulcerated forms (median 45 days, IQR 30–105 versus 30 days, IQR 30–60, respectively, with p = 0.03) (S1 Table). Even when age and gender were adjusted in binary logistic regression, we observed an increased risk of developing ulcerative lesions as each day/month passed (Table 4).

**Table 4:** Multivariate analysis of the effect of time-delay on clinical BU forms in 476 laboratory-confirmed BU treated patients at CDTUB—Allada from 2005 to 2013 (binary logistic regression).

	Coeff. (SE)	Odds Ratio (CI) (days)	Odds Ratio (CI) (months)	p-value
***Male***				
Age	0.006	0.973 (0.961–0.985)		**0.0001**
Time delay ^a^	0.001	1.000 (0.999–1.001)	0.999 (0.966–1.034)	0.799
***Upper body lesions*** ^b^				
Age	0.006	0.987 (0.975–0.999)		**0.028**
Gender	0.192	0.837 (0.574–1.218)		0.352
Time delay	0.001	0.999 (0.998–1.001)	0.980 (0.945–1.017)	0.299
***Ulcerative lesions***				
Age	0.006	1.002 (0.989–1.014)		0.793
Gender	0.204	1.067 (0.709–1.576)		0.786
Time delay	0.001	1.002 (1.000–1.004)	1.065 (1.005–1.129)	**0.025**

^a^ time delay until seeking medical care; odds ratio for days and months

^b^ upper body lesions: head + neck + upper limbs + thorax + abdomen

**Fig 6:**
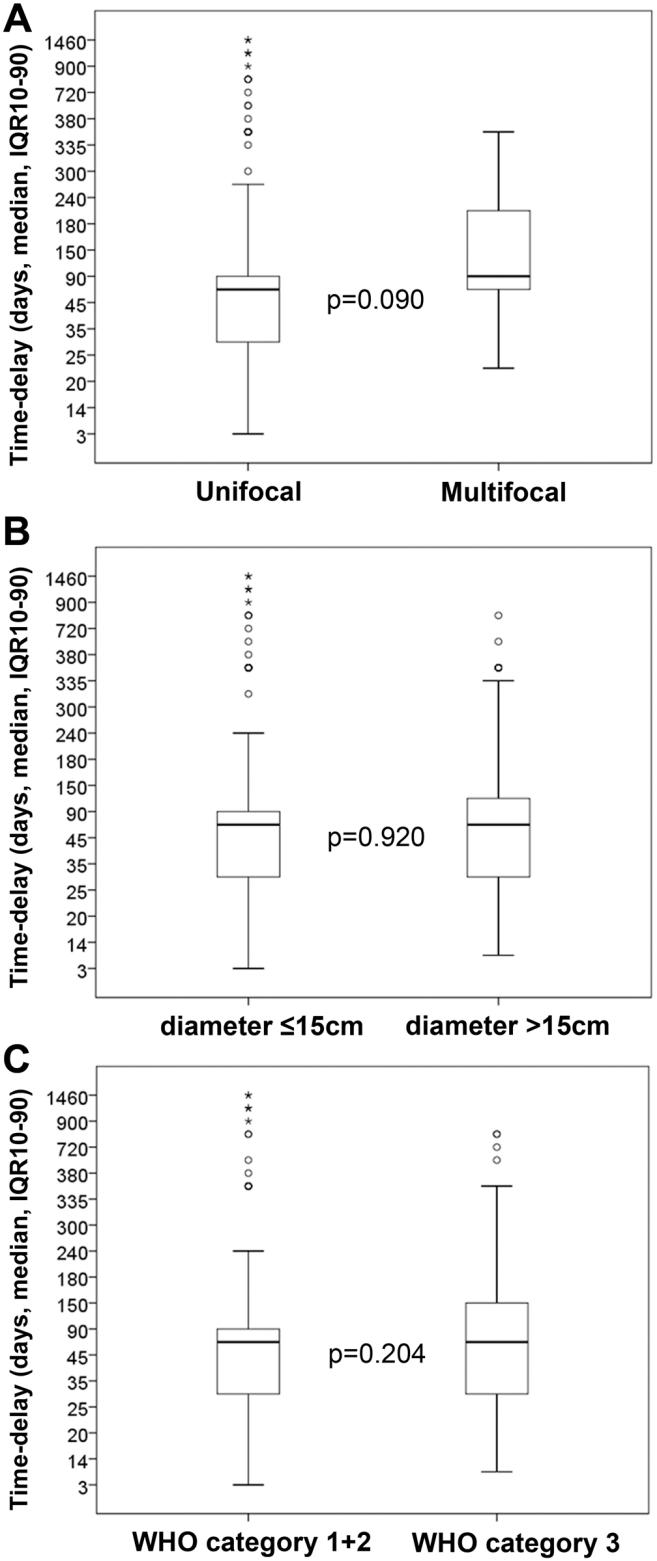
Time-delay to seek medical care related to severe BU forms. (A) multifocality (multifocal vs. unifocal lesions); (B) lesion size (≤15cm vs. >15cm); (C) WHO Category (Category 3 vs. category 1+2). Circles represent the outliers and asterisks represent the extreme outliers. Statistical significance was calculated using Welch's *t*-test. Differences with a p-value of ≤0.05 were considered significant.

Considering severe forms of BU, none of the aggressive phenotypes were considered related to significantly different delay times to seek medical care: multifocal lesions (median 90 days, IQR 56.3–217.5, p = 0.09) (Fig 6A and S2 Table), larger lesions with diameter >15cm (median 60 days, IQR 30–120, p = 0.92) (Fig 6B and S2 Table) or category 3 WHO classification (median 60 days, IQR 30–150, p = 0.20) (Fig 6C and S2 Table), when compared with unifocal (median 60 days, IQR: 30–90), small lesions (diameter ≤15cm) (median 60 days, IQR 30–90) or WHO category 1+2 lesions (median 60 days, IQR 30–90), respectively (Fig 6A–6C and S2 Table). Finally, when systematically fit within binary (dichotomized variables) (Table 5) or linear (Table 6) logistic regression models controlling for age and gender, time-delay to seek medical care remained statistically insignificant with respect to the occurrence of the most aggressive severe clinical forms.

**Table 5:** Multivariate analysis of the effect of time-delay on severe BU forms in 476 laboratory-confirmed BU treated patients at CDTUB—Allada from 2005 to 2013 (binary logistic regression).

	Coeff. (SE)	Odds Ratio (CI) (days)	Odds Ratio (CI) (months)	p-value
***Multifocal lesions***				
Age	0.013	1.009 (0.984–1.034)		0.48
Gender	0.452	1.203 (0.496–2.918)		0.68
Time delay ^a^	0.001	1.001 (0.999–1.003)	1.032 (0.978–1.089)	0.26
***Larger lesions (***>***15 cm)***				
Age	0.006	1.004 (0.992–1.016)		0.536
Gender	0.211	1.462 (0.967–2.208)		0.071
Time delay	0.001	1.000 (0.999–1.001)	0.980 (0.945–1.017)	0.925
***WHO category 3***				
Age	0.006	1.003 (0.991–1.015)		0.652
Gender	0.203	1.429 (0.960–2.128)		0.079
Time delay	0.001	1.001 (1.000–1.002)	1.020 (0.961–1.035)	0.247

^a^ time delay until seeking medical care; odds ratio for days and months

**Table 6:** Multivariate analysis of the effect of time-delay on severe BU forms in 476 laboratory-confirmed BU treated patients at CDTUB—Allada from 2005 to 2013 (linear model regression).

	B and 95%CI	p-value
***Lesion Size***		
Age	0.001 (-0.070–0.072)	0.977
Gender	1.369 (-0.867–3.605)	0.229
Time delay ^a^	(-)0.002 (-0.009–0.005)	0.598
***Category 1*, *2 and 3 (WHO)***		
Age	0.001 (-0.002–0.003)	0.657
Gender	0.079 (-0.009–0.166)	0.079
Time delay	0.0001 (0.0001–0.0001)	0.244

^a^ time delay until seeking medical care; odds ratio for days and months

## Discussion

BU pathogenesis is related with necrosis of the subcutaneous tissue associated with mycolactone, the potent cytotoxic/immunosuppressive toxin produced by *M*. *ulcerans* [3]. Initial pre-ulcerative lesions (papules, nodules, plaques and edematous infiltration) can evolve into ulcers and progressively spread over significant extensions of the body [9] or even affect the bone [10]. Large national studies in West African countries, namely Ghana [31], Benin [28,29,32] and Côte D`Ivoire [33], included the largest BU cohorts studied thus far and provided information about the age and gender of patients, site of lesions and the major clinical forms—providing further clues on the evolution of BU pathology. The majority of these studies used distinct methodologies (retrospective and/or prospective cohorts; cross-sectional) and a descriptive approach, with a large proportion of diagnoses being retrospective and scar-based. Here, we strictly consider laboratory-confirmed BU patients.

Concerning the BU clinical forms (papules, nodules, plaques, edematous infiltration, ulcers and osteomyelitis), the observations of our study globally fit the variances reported in those larger cohorts. Specifically, we confirm that BU is mainly a paediatric disease (median age of diagnosis 12 years with IQR: 7–24 years and mean of 19.7 years); with a predominance of lesions on the lower limbs (53.8%); a predominance of ulcerative forms (67.2%); and with an equilibrium between genders. In addition, there is a distinct distribution of gender when age is considered, with males being overrepresented in younger patients, reproducing data from previous studies [15,29].

Osteomyelitis and edematous forms are classified as belonging to the spectrum of BU presentations, although some authors consider them to be more severe clinical forms [32,34,35,36]. Regarding osteomyelitis, a great variance in prevalence is described and further complexity is added when suspected non-confirmed cases of bone involvement are included in the analysis. Indeed, reported prevalence values of bone disease related to BU were as high as 29.5% [37] and 36.1% [38]. However, when only confirmed osteomyelitis cases were considered, prevalence decreased with values ranging between 6% [29] and 20% [39] in Africa and only 1% in Australia [40]. Moreover, HIV infection seems to favour the occurrence of osteomyelitis [17]. In the present study, osteomyelitis lesions only occurred in 1.1% of the at-risk population, which could be related to the fulfillment of confirmed diagnosis criteria (e.g. x-ray or surgical evidence) and the absence of the HIV co-infection selection criteria.

Eedematous lesions manifest as diffuse, extensive, usually non-pitting swelling with ill-defined margins involving part or all of a limb or other part of the body [41]. Cases of edematous *M*. *ulcerans* infection can be misdiagnosed as bacterial cellulitis leading to delays in diagnosis, progression of disease, increased morbidity and increased complexity and cost of treatment. Additionally, edema is often self-perceived as not being a relevant health problem, therefore delaying seeking medical attention. In previous studies, prevalence was determined to be between 2.5% [42] and 12.5% [31]. In our study, edematous forms accounted for 5% of the studied population, fitting with the prevalence reported in similar cohorts [31,34,35,36,42].

Within to the above described clinical BU presentations, more aggressive, severe clinical presentations have been described [11], although the underlying pathological mechanisms are yet unclear [29]. In our study, within the severe phenotypes, 33.8% of the patients were in WHO category 3; 4.5% presented multifocal forms; and 29.8% of the patients presented lesions >15cm in major diameter. Regarding multifocal lesions, previous studies describe highly variable prevalences (e.g. 2.0% -11.1%) [40,42,43,44,45,46]. Moreover, in our African cohort, we verify that age does not associate with multifocal lesions, conversely to an Australian cohort [40]. Regarding lesion size, only a few studies report large lesions as a specified studied variable, since these lesions are usually included in category 3 lesions. However, when considered separately, their prevalence ranged between 11.1% [47] and 36.0% [29], while category 3 lesions have been reported to range between 19.7% [48] and 60.0% [39]–values replicated in the present study.

The effect of time-delay in seeking medical care for BU patients is a relevant issue for public health and patient management. Our observations in a cohort of laboratory-confirmed cases of BU show that gender was not related with distinct behavior in seeking specific medical care and that younger patients, mainly through their parents/legal tutors, spent less time seeking medical attention prior to diagnosis (median 45 versus 60 days, for the group ≤15 years old versus >15 years old respectively, p = 0.004). In line with previous African studies, we found that more advanced ulcerative forms were related to the delay in seeking medical care. Remarkably, and contrary to what one would expect, we found that multifocal lesions, larger lesions or WHO category 3 lesions may be considered distinct clinical entities since the time-delay in seeking medical attention had no significant role in disease progression. As a matter of fact, in Africa, time-delay was seen as a marker of accessibility to medical care and, in fact, some studies compare time-lapse before and after interventional politics on health care improvement. In West Africa, studies reported a time-delay between 42 [38] and 84 days [44,49], taking into consideration all clinical forms. Specifically, a Beninese study reported distinct clinical forms relating to time-lapse since first symptoms were remembered [28]. Time-delay was shorter for non-ulcerated clinical forms (median 30 to 46 days), than for ulcerated forms (median 61 days) and larger for osteomyelitis (median 91 days).

In Australian studies, time-lapse until medical care was reported to be much shorter—between 14 days (IQR 0–6 weeks) [40] and 42 days (ranging from 2 and 270 days) [50]. In this distinct health-care reality, determinants for delay in seeking medical care were related to atypical sites of lesions, associated with an increased complexity in medical BU diagnosis. Interestingly, in Australian patients, ulcerated versus non-ulcerated clinical forms did not experience significantly different time lapses. Moreover, independently of the advances in diagnosis and clinical management, there was no variation in time-delay between 1998–2004 and 2005–2011.

In Southern America, the time-delay reported among Peruvian BU patients was between 1 and 8 months [51].

Overall, our observations in a cohort of laboratory-confirmed cases of BU, strengthening previous observations and show that the time-delay in seeking medical care is related to the more advanced ulcerative forms, further justifying early diagnosis and treatment. Notably, we additionally show that time-delay was not significantly associated with more severe phenotypes of BU, such as multifocal lesions, larger lesions or WHO category 3 lesions. Indeed, our results demonstrate that after initial progression lesions become stable regarding size and focal/multifocal progression. Therefore, in future studies on BU epidemiology, severe clinical forms should be systematically considered as distinct phenotypes of the same disease and therefore subjected to specific risk factor investigation. These results further highlight that intrinsic regulatory mechanisms, such as the host immune response and local biochemical and physical factors, most likely have relevant roles in determining severe phenotypes, justifying more structural immune-related and bacterial genetic studies.

## Supporting Information

S1 Table Time-delay according to gender, age, lesion location and classical lesion phenotype in 476 laboratory-confirmed BU treated patients at CDTUB—Allada from 2005 to 2013. (DOCX)

S2 Table Time-delay according to gender, age, lesion location and severe lesion phenotype in 476 laboratory-confirmed BU treated patients at CDTUB—Allada from 2005 to 2013. (DOCX)

S1 Checklist STROBE checklist. (DOCX)
