# Supplementary material for: Clinical Epidemiology of Buruli Ulcer from Benin (2005-2013): Effect of Time-Delay to Diagnosis on Clinical Forms and Severe Phenotypes
Source: PLoS Negl Trop Dis. 2015 Sep 10;9(9):e0004005. doi: 10.1371/journal.pntd.0004005 (PMC4565642; doi:10.1371/journal.pntd.0004005)
Supplement: S1 Table — (DOCX) [file pntd.0004005.s001.docx]

**S1 Table**. Time-delay according to gender, age, lesion location and clinical lesion phenotype in 476 laboratory-confirmed BU treated patients at CDTUB - Allada from 2005 to 2013.

|  | **Time-delay** ^b^  **days median [IQR]** | **Time-delay** ^b^  **days mean (95%CI)** | **p-value** ^c^ |
| --- | --- | --- | --- |
| ***Gender*** |  |  |  |
| Male | 60 [30-90] | 96.6 (78.1-114.5) | p=0.538 |
| Female | 60 [30-90] | 106.04 (81.7-130.4) |  |
| ***Age*** |  |  |  |
| > 15 years old | 60 [30-120] | 140.1 (103.1-177.1) | **p=0.004** |
| ≤ 15 years old | 45 [30-90] | 82.5 (69.8-95.2) |  |
| ***Lesion location*** ^a^ |  |  |  |
| Head and neck | 52.5 [30-90] | 68.0 (2.8-133.2) | p=0.614 |
| Thorax and abdomen | 60 [30-120] | 103.4 (64.6-142.2) |  |
| Upper Limb | 60 [30-90] | 89.2 (67.8-110.5) |  |
| Lower Limb | 60 [30-90] | 109.5 (86.7-132.2) |  |
| Lower limb lesions vs. Upper limb lesions | 60 [30-90] | 109.5 (86.7-132.2) | p=0.233 |
|  | 60 [30-90] | 91.4 (73.2-109.5) |  |
| ***Clinical lesion*** |  |  |  |
| Nodule ^a^ | 30 [25-75] | 50.3 (21.0-120.0) |  |
| Edema ^a^ | 45 [30-105] | 84.2 (50.1-126.3) |  |
| Plaque ^a^ | 30 [30-60] | 73.9 (50.1-109.5) |  |
| Ulcer ^a^ | 60 [30-120] | 111.0 (92.9-130.9) |  |
| Ostemyelitis ^a^ | 365 [228-548] | 395.0 (90.0-730.0) |  |
| All | 60 [30-90] | 101.1 (86.3-117.0) |  |
| Non-ulcerative vs. Ulcerative forms | 32.5 [30-67.5] | 81.1 (55.3-106.9) | **p=0.009** |
|  | 60 [30-120] | 111.0 (92.9-130.9) |  |
| Edema vs. Other non-ulcerated forms | 45 [30-105] | 84.2 (50.1-126.3) | **p=0.030** |
|  | 30 [30-60] | 73.1 (44.7-101.5) |  |

^a^ dominant clinical form

^b^ time delay until seeking medical care

^c^ comparison of median - time delay distribution between groups with Welch's t-test.
