# Supplementary material for: Clinical Epidemiology of Buruli Ulcer from Benin (2005-2013): Effect of Time-Delay to Diagnosis on Clinical Forms and Severe Phenotypes
Source: PLoS Negl Trop Dis. 2015 Sep 10;9(9):e0004005. doi: 10.1371/journal.pntd.0004005 (PMC4565642; doi:10.1371/journal.pntd.0004005)
Supplement: S2 Table — (DOCX) [file pntd.0004005.s002.docx]

**S2 Table**. Time-delay according to gender, age, lesion location and severe lesion phenotype in 476 laboratory-confirmed BU treated patients at CDTUB - Allada from 2005 to 2013.

|  | **Time-delay** ^a^  **days median [IQR]** | **Time-delay** ^a^  **days mean (95%CI)** | **p-value** ^b^ |
| --- | --- | --- | --- |
| ***Multifocal lesions*** |  |  |  |
| Multifocal | 90 [56.3-217.5] | 141.9 (94.1-189.7) | p=0.090 |
| Unifocal | 60 [30-90] | 99.1 (83.7-114.5) |  |
| ***Lesion size*** |  |  |  |
| ≥ 15cm | 60 [30-120] | 102.1 (83.8-120.4) | p=0.920 |
| < 15cm | 60 [30-90] | 100.7 (81.1-120.4) |  |
| ***WHO Category*** |  |  |  |
| Category 3 | 60 [30-150] | 113.4 (92.9-133.9) | p=0.204 |
| Category 1 + 2 | 60 [30-90] | 95.0 (75.2-114.8) |  |

^a^ time delay until seeking medical care

^b^comparison of median - time delay distribution between groups with Welch's t-test.
